# Humanoid Robot Walking and Grasping Method Using Similarity Reward-Augmented Generative Adversarial Imitation Learning

**DOI:** 10.3390/s26092756

**Published:** 2026-04-29

**Authors:** Gen-Yong Huang, Wen-Feng Li

**Affiliations:** School of Transportation and Logistics Engineering, Wuhan University of Technology, Wuhan 430063, China; 17307008081@163.com

**Keywords:** humanoid robot, generative adversarial imitation learning, similarity reward, walking-grasping coordination, maximum entropy reinforcement learning

## Abstract

This study aims to enhance the precision of humanoid robots in imitating complex human “walking–grasping” coordinated movements. Addressing limitations in sample efficiency and reward function design in Generative Adversarial Imitation Learning (GAIL), we propose the Similarity Reward-Augmented Generative Adversarial Imitation Learning (SRA-GAIL) framework. The method integrates plantar thin-film resistive pressure sensors to measure the real-time pressure distribution at four key points on both feet, combined with roll/pitch angle data acquired from JY901S inertial measurement units (IMUs). A Lagrangian constraint optimization strategy is employed to achieve gait stability control based on the zero moment point (ZMP). Simultaneously, a visual similarity evaluation module is established using human demonstration trajectories captured by a Logitech C920E camera, augmented by grip force feedback from flexible thin-film pressure sensors on the hands. This enables the design of a multimodal sensor-fused similarity reward function. By incorporating Lagrangian constraint optimization and a maximum entropy reinforcement learning framework, Similarity Reward-Augmented Generative Adversarial Imitation Learning synchronously optimizes gait stability control—guided by zero moment point (ZMP) and roll/pitch data—and vision-based trajectory similarity evaluation. These components address motion stability constraints and trajectory similarity metrics, respectively, generating biomechanically plausible gait strategies. A spatiotemporal attention mechanism parses human motion trajectory features to drive the end-effector for high-precision trajectory tracking. To validate the proposed method, an imitation learning experimental system was constructed on a physical XIAOLI humanoid robot platform, integrating inertial measurement units (IMUs), plantar pressure sensors, and a vision system. Quantitative evaluations were conducted across multiple dimensions, including robot platform analysis, walking stability, object grasping success rates, and end-effector trajectory similarity. The results demonstrate that, compared to Generative Adversarial Imitation Learning (GAIL) and behavioral cloning, Similarity Reward-Augmented Generative Adversarial Imitation Learning achieves a stable object grasping success rate of 93.7% in complex environments, with a 23.8% improvement in sample efficiency. The method maintains a 96.5% compliance rate for zero moment point (ZMP) trajectories within the support polygon, significantly outperforming baseline approaches. This effectively addresses the bottleneck in robot policies adapting to dynamic changes in real-world environments.

## 1. Introduction

The locomotor capabilities of humanoid robots in walking and grasping tasks are fundamental for their execution of complex operations in dynamic environments. Researchers have drawn inspiration from biomechanics, leveraging imitation learning to transfer human motion strategies to robot control, thereby enhancing adaptability and flexibility [1,2,3,4,5]. For instance, Liang et al. [6] introduced a multimodal reinforcement learning framework that fuses visual and force-sensing data as state inputs, facilitating the acquisition of dexterous multifinger manipulation skills. Behrens et al. [7] utilized motion trajectories captured by wearable devices alongside visual positioning information to develop a demonstration-based learning method for dual-arm robots, enabling the planning of complex grasping paths. In deep reinforcement learning, Zakka et al. [8] optimized robotic precision assembly tasks through scene object representation extraction, highlighting the critical role of sensor data fusion in perception–control loops. Generative Adversarial Imitation Learning (GAIL), as a key branch of imitation learning [9], learns policies directly from expert demonstrations via adversarial interplay between a generator and a discriminator. This approach bypasses the intricate intermediate steps of inverse reinforcement learning, significantly improving the learning efficiency [10]. The core idea involves optimizing a reward function to assign high values to expert state–action pairs. However, due to the non-differentiability of policy models in action spaces, the high-dimensional stochasticity of humanoid dynamics, and the sample efficiency reliance of model-free reinforcement learning, conventional Generative Adversarial Imitation Learning methods often encounter challenges such as reward function ambiguity and policy instability in real-world scenarios [11,12,13]. Particularly in walking and grasping tasks, single-modality sensor data struggle to comprehensively capture the dynamic features of environmental interactions, limiting the policy generalization capabilities. Peng et al. [14] trained single-task policies using unlabeled action data but exhibited constraints in multiskill integration contexts. Chebotar et al. [15] achieved the conditional generation of multimodal policies by mapping motion data and text labels into a latent space. Wirth et al. [16] employed reward functions based on substantial human feedback to train control policies, ensuring high similarity between robot behaviors and human demonstrations. Christiano et al. [17] transformed human feedback into reward signals for reinforcement learning via query-based learning. Nonetheless, existing studies still exhibit room for improvement in sample efficiency. For example, Haarnoja et al. [18] developed reinforcement learning algorithms capable of optimization but required prolonged training periods. Ma et al. [19] proposed a framework based on traditional adversarial latent models, utilizing conditional discriminators to match latent variables with actions. Polydoros et al. [20] extended generative adversarial networks’ applications in imitation learning, but their conditional discriminators primarily served for trajectory annotation rather than reward generation, constraining further gains in sample efficiency.

To address this challenge, we propose the Similarity Reward-Augmented Generative Adversarial Imitation Learning (SRA-GAIL) method. This approach innovatively integrates similarity rewards with multisensor data streams. Specifically, thin-film resistive pressure sensing units mounted on the feet measure pressure at four endpoints of both feet, and the zero moment point (ZMP) is analyzed to optimize walking stability. The JY901S inertial measurement unit (IMU) is employed to acquire roll and pitch angle data for attitude estimation, while a Logitech C920E camera captures human trajectories to compute motion similarity rewards, thereby enhancing the bionic nature of policy learning. Additionally, the FSR-GT sensor monitors the hand grip force, improving the force control accuracy in grasping tasks. Similarity Reward-Augmented Generative Adversarial Imitation Learning optimizes the reward function via Lagrangian constraints and incorporates a maximum entropy reinforcement learning algorithm, enabling the robot policy to progressively approximate the optimal trajectory of human demonstrations while satisfying real-time constraints. Experiments are conducted using an integrated imitation learning system comprising a graphics workstation, software algorithms, and the XiaoLi humanoid robot, with validation performed on the Panoptic Studio [10] motion dataset. The main contributions of this study include (1) a novel sensor-based similarity reward method to enhance the multimodal data fusion capabilities of generative adversarial imitation learning; (2) a comprehensive sensor data preprocessing pipeline designed to improve the walking stability, grasping accuracy, and trajectory similarity; and (3) systematic experimental validation demonstrating the superior performance of the proposed method in complex environments.

## 2. Methods

**Figure 1:**
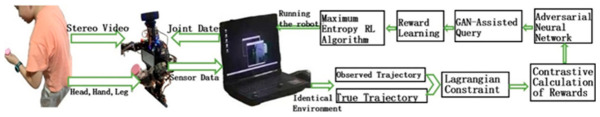
Generative Adversarial Imitation Learning method based on similarity reward.

This study proposes a reinforcement learning method enhanced by human motion similarity based on generative adversarial networks, termed Similarity Reward-Augmented Generative Adversarial Imitation Learning (SRA-GAIL). As illustrated in Figure 1, the method generates a reward signal by comparing the similarity between human and robot motion trajectories, which is then used to optimize the policy learning process via reinforcement learning algorithms. The core of the Generative Adversarial Imitation Learning (SRA-GAIL) framework is adversarial optimization. It constructs an environmental model based on the Markov decision process (MDP) for trajectory estimation and optimization. Specifically, the environment is modeled as a tuple $\left(S,A,T,V,\gamma ,y_{0}\right)$, where S denotes the state space, A the action space, T the time step, γ∈(0,1) the discount factor, r the reward function, and $y_{0}$ the initial state distribution. The policy π(a|s) induces a trajectory distribution over state–action pairs.

At each time step t, the robot generates an action trajectory segment $\left(\xi^{real},\xi^{moni}\right)$ according to the current policy π. The robot receives observations $s_{t}\in S$ from the environment and produces actions $a_{t}\in A$ to interact with it, facilitating trajectory similarity evaluation. A discriminative neural network model *X* is trained to learn the selection probability of human sample trajectories. Let the trajectory segment $\xi_{i}=[s_{i+1},a_{i+1},s_{i+2},a_{i+2},\ldots ,s_{i+k},a_{i+k}]$ represent similar trajectories selected from a sample trajectory set ξ. The model parameters θ are updated via a stochastic gradient ascent algorithm. By acquiring the robot’s state information, control commands, and clock data at time step t, the method learns human sample trajectories $\xi =[s_{1},a_{1},s_{2},a_{2},\ldots ,s_{k},a_{k}]$. The similarity value of the trajectory segment is computed as a reward signal for RL, guiding the robot to generate behaviors that more closely approximate genuine human trajectories.

Through the integration of sensor data with the similarity reward mechanism, the generated trajectories exhibit enhanced smoothness and accuracy, with action execution more closely resembling human operator characteristics. Key state information, such as the robot’s actual foot placement and lift height, is incorporated into the observations as critical observables to improve the trajectory quality and naturalness. During policy evaluation, the similarity value SIM:S∗A→r is estimated from the trajectory segment $\left(\xi^{real},\xi^{moni}\right)$, and the result is stored in the trajectory segment $\xi_{i}=[s_{i+1},a_{i+1},s_{i+2},a_{i+2},\ldots ,s_{i+k},a_{i+k}]$ in the form of a triple $\left(\xi^{real},\xi^{moni},\theta \right)$. This method achieves adaptive optimization in dynamic environments, enhancing the consistency, flexibility, and generalizability of robot behaviors. By learning intertrajectory differences, it effectively distinguishes between real human trajectories and robot-generated trajectories, thereby improving the robustness and performance in practical applications.

### 2.1. Similarity Analysis

In action similarity analysis, r is regarded as a latent factor explaining human decision-making processes, and it is hypothesized that the similarity probability of action segments $\xi_{i}$ exhibits an exponential relationship with the cumulative implicit reward $Y(s_{t},a_{t})=\sum_{i}r(s_{t+i},a_{t+i})$, thereby capturing the similarity characteristics between them and the reference action $Y(s_{t},a_{t})$. By utilizing real human trajectory data to supervise the training of the discriminator network, the optimal approximation of individual state–action pairs is achieved under the Generative Adversarial Imitation Learning framework. Additionally, the similarity increment of the robot’s position is computed and joint torques are adjusted to minimize the cross-entropy loss *L* between human and robot actions, expressed as

(1) $$L(\pi^{real},\pi^{moni},\theta )=E_{S_{t},A_{t},\ldots ,S_{n},A_{n}}\sum_{k=t}^{n}\ln[1-D(s_{k}^{real},a_{k}^{real};\theta )]+E_{S_{t},A_{t},\ldots ,S_{n},A_{n}}\sum_{k=t}^{n}\ln[1-D(s_{t}^{moni},a_{t}^{moni};\theta )]$$

where $E_{S_{t},A_{t},\ldots ,S_{n},A_{n}}$ denotes the expected value, $\xi^{moni}$ represents data from the observed generated trajectories, $\xi^{real}$ is the robot’s operational trajectory, D is the predefined discriminator, and (s,a) defines the state–action pair.

The objective of generative adversarial learning is to construct models capable of actively learning optimal human behaviors. Thus, the model’s policy network is updated through adversarial training. The parameters *θ* of the current policy network *π* are evaluated by optimizing the behavioral similarity, which primarily relies on the matching degree between state–action pairs generated from observed trajectories and those from real trajectories. In the Similarity Reward-Augmented Generative Adversarial Imitation Learning (SRA-GAIL) framework, the policy network parameters *π* are optimized via adversarial training with the discriminator. The core of behavioral similarity assessment lies in comparing the state–action pairs of generated trajectories with those of real trajectories. The discriminator must distinguish between trajectory state–action pairs produced by the human observation generator $\xi^{real}=[s_{1}^{real},a_{1}^{real},s_{2}^{real},a_{2}^{real},\ldots ,s_{s}^{real},a_{s}^{real}]$ and those from the robot’s execution trajectory $\xi^{moni}=[s_{1}^{moni},a_{1}^{moni},s_{2}^{moni},a_{2}^{moni},\ldots ,s_{k}^{moni},a_{k}^{moni}]$. Consequently, Similarity Reward-Augmented Generative Adversarial Imitation Learning quantifies the proximity between the two through a similarity reward mechanism. When the discriminator’s output score *π* approaches 1, it indicates high similarity between the generated and real trajectories; conversely, a score near 0 signifies substantial differences. To enhance the precision of action feedback, the trajectory position difference serves as the basis for calculating the similarity reward. The coordinate state–action pairs of real human trajectories $\xi^{real}=[s_{1}^{real},a_{1}^{real},s_{2}^{real},a_{2}^{real},\ldots ,s_{s}^{real},a_{s}^{real}]$ are acquired via visual sensors, while the state–action pairs of robot trajectories $\xi^{moni}=[s_{1}^{moni},a_{1}^{moni},s_{2}^{moni},a_{2}^{moni},\ldots ,s_{k}^{moni},a_{k}^{moni}]$ are obtained through joint encoders. The discriminator network is supervised using real human trajectory data to differentiate between generated and real trajectories. In the Similarity Reward-Augmented Generative Adversarial Imitation Learning method, the policy network optimizes the strategy by minimizing the cross-entropy loss between robot and human actions, training with full-time state–action pairs of the robot. The discriminator and policy model adversarially update the next human state information to achieve the homogenization of human and robot trajectory similarity r=D(s,a). The trajectory position difference provides precise feedback for each action step by computing the distance difference between the robot trajectory point and the human trajectory point at each time instant as a position reward $r_{P}$, calculated as

(2) $$r_{P}^{t}=\frac{1}{1-\exp\left(\sum_{i}\overset{\wedge }{r}\left(s_{t+i}^{real},a_{t+i}^{real}\right)-\overset{\wedge }{r}\left(s_{t+i}^{moni},a_{t+i}^{moni}\right)\right)}t$$

where t represents the current time instant, $s_{t+i}^{real}$, $a_{t+i}^{real}$ denotes the coordinates of the human trajectory captured at time t, and $s_{t+i}^{moni}$, $a_{t+i}^{moni}$ represents the coordinates of the robot trajectory at time t. This position reward drives joint actions, facilitating the robot in performing walking and grasping tasks.

In terms of velocity and acceleration matching optimization, the mean and standard deviation of the velocity and acceleration are statistically derived from human demonstration data, with β serving as a constraint adjustment coefficient. When the robot’s velocity or acceleration falls within the range of the mean β times the standard deviation, a reward enhancement mechanism is applied. Simultaneously, the product of the velocity and acceleration distributions of various actions from real human data and the coefficient is subtracted from the reward, specifically computed as

(3) $$r_{e}=\frac{\left|\upsilon_{t}-\upsilon_{mean}\right|}{\beta \upsilon_{std}}$$

where $r_{e}$ is the efficiency reward, $\upsilon_{mean}$ denotes the average velocity of the robot, $\upsilon_{std}$ is the standard deviation of all robot velocities, and β is an adjustment coefficient determined through parameter optimization.

For safety constraints in walking and grasping tasks, a safety protection mechanism based on elliptical regions is designed. The left and right foot landing points of the robot define semi-elliptical forbidden zones, triggering safety penalties when obstacles intrude. The safety distance calculation relies on measurements from foot-end force sensors, where θ represents the distance between the foot-end and the obstacle. This constraint is incorporated into the optimization objective using Lagrange multipliers λ through the real-time monitoring of ground contact states via foot-end force sensors:

(4) $$\begin{array}{l} g\left(\theta \right):\sum_{t=1}^{T}r_{s}^{t}-r_{\lim it}<0\\ \underset{\theta }{\max}\;\underset{\lambda \geq 0}{\min}L\left(\theta \right)=f\left(\theta \right)-\lambda \left(\theta \right) \end{array}$$

where $g\left(\theta \right)$ defines the constraint condition, T is the maximum walking and grasping step length, $\sum_{t=1}^{T}r_{s}^{t}$ is the cumulative value of safety constraints, $r_{\lim it}$ denotes the threshold of safety constraints, and $L\left(\theta \right)$ is the optimization objective. An efficiency reward $f\left(\theta \right)$ is introduced to enhance action smoothness constraints, with λ as the Lagrange multiplier. Model parameters are updated via gradient ascent $\pi^{*}(a_{t+1}|s_{t+1})$, and $\lambda \left(\theta \right)$ is updated via gradient descent. In practical implementation, foot-end force sensors monitor environmental states in real time, mapping obstacle distances to the safety penalty mechanism. Sensor data are preprocessed and associated with the constraints of Formula (4), ensuring that the robot effectively avoids risks while imitating human trajectories, thereby improving the physical safety.

### 2.2. Training Reward Function

During the interaction between the robot and the environment, the robot acquires environmental state information s through a multimodal sensor array comprising cameras, inertial measurement units (IMUs), force-sensitive resistors (FSRs), and grasping force sensors. Based on this information, the robot selects and executes corresponding walking and grasping operations a. The state-to-action mapping is defined by a policy model $\pi_{\theta }(a|s)$, where the policy parameters π are denoted as θ. According to the state transition equation $y(s_{t+1}|s_{t},a_{t})$, the robot transitions to the next state and receives a reward value $r_{t}=r^{*}(s_{t},a_{t},\pi )$ for the corresponding action, aiming to maximize the sum of cumulative discounted rewards. The optimal policy $\pi^{*}$ is computed as follows:

(5) $$\pi^{*}=\arg\;\underset{\pi }{\max}\left[\sum_{k=t}^{n}E_{S_{t},A_{t},\ldots ,S_{n},A_{n}}\left[\gamma^{k-t}\cdot r^{*}(s_{t},a_{t},\pi )\right]\right]$$

A comprehensive reward function r is designed to enhance the imitation learning performance by integrating multidimensional information. This reward function consists of three components: a behavioral similarity reward, a positional reward, and an efficiency constraint. The robot utilizes the multimodal sensor array, including cameras, IMUs, FSRs, and force sensing, to obtain the environmental state space S and action space A. From the set of human-selected sample trajectories, the robot learns to select the optimal trajectory set, and the integrated reward function r is formulated as

(6) $$r=\omega_{G}r_{G}+\omega_{P}r_{P}+\omega_{e}r_{e}$$

In the equation, $r_{G}$ represents the behavioral similarity reward, which quantifies the similarity between the robot’s actions and those of human experts; $r_{P}$ denotes the positional reward, reflecting the deviation between the robot’s current state and the target position; and $r_{e}$ signifies the efficiency constraint term, ensuring task completion while satisfying time or energy consumption efficiency requirements. $\omega_{G}$, $\omega_{P}$, $\omega_{e}$ correspond to the weight coefficients for the robot’s pose relative to the target position.

### 2.3. Training of Maximum Entropy Reinforcement Learning Algorithm

During the training process, the robot samples from a mixed experience replay buffer composed of expert demonstration trajectories and automatically generated trajectories to achieve the effective exploration of the state space S and action space A. The experience buffer is constructed based on human demonstration data, and training is conducted using the maximum entropy reinforcement learning algorithm. After obtaining the reward estimate r, the maximum entropy reinforcement learning method is employed to maximize the expected return $\pi^{*}$ by leveraging human learning strategies, thereby establishing the maximum entropy reinforcement learning formulation as follows:

(7) $$\pi^{*}=\arg\;\underset{\pi }{\max}\left[\sum_{k=t}^{n}E_{\xi_{\omega }}\left[\gamma^{k-t}\cdot R^{*}(s_{t},a_{t},\theta )\right]-E_{\xi_{n\omega }}\log\left[\gamma^{k-t}\cdot R^{*}(s_{t},a_{t},\theta )\right]\right]$$

The maximum entropy reward function based on a stochastic policy is defined as

(8) $$r^{*}(s_{t},a_{t},\pi )=\overset{\wedge }{r}(s_{t},a_{t})+\beta H_{p}(\pi (\cdot |s_{t}))$$

where the parameter β is used to determine the maximum entropy form $H_{p}(\pi (\cdot |s_{t}))$ of the reward function $r^{*}(s_{t},a_{t},\pi )$. The similarity-based policy learning problem can be transformed into the following optimization form:

(9) $$\pi^{*}=\arg\;\underset{\pi }{\max}\left[\sum_{k=t}^{n}E_{\xi_{\omega }}\left[\gamma^{k-t}\cdot \overset{\wedge }{r}(s_{t},a_{t})+\alpha H_{p}(\pi (\cdot |s_{t}))\right]-E_{\xi_{n\omega }}\log\frac{\exp\left[\gamma^{k-t}\cdot \overset{\wedge }{r}(s_{t},a_{t})+\alpha H_{p}(\pi (\cdot |s_{t}))\right]}{\pi (s_{t+i}|a_{t+i})}\right]$$

Compared to Equation (7), Equation (9) introduces a novel logarithmic term that learns the optimal expectation of similarity-based human motion trajectory pairs from the objective function. This term takes the robot’s proprioceptive state and environmental interaction state as inputs and incorporates robot constraints into the optimization objective via the Lagrangian method. By performing similarity matching with pre-stored human expert trajectories, the optimization objective for policy updates is defined in f(θ) within Equation (4), while g(θ) analogously computes the safety constraint penalty network. Proximal policy optimization (PPO) is employed to update the policy function π, optimizing policy parameters θ by balancing the trust region between the new and old policies. This ensures that training relies on real-time feedback from sensors, including data such as joint angles, accelerations, contact forces, and object poses, which directly influence the trajectory similarity evaluation and policy optimization efficacy. The adversarial neural network requires human-like similarity to generate plausible data points; thus, the objective function can be formulated as follows:

(10) $$L\left(\theta \right)=\sum_{k=t}^{n}\left\{E_{\xi_{\omega }}\left[\gamma^{k-t}\overset{\wedge }{R}(s_{t},a_{t})+\alpha H_{p}(\pi_{\theta }(\cdot |s_{t}))\right]-E_{\xi_{n\omega }}\log\left(\frac{\exp\left[\gamma^{k-t}\overset{\wedge }{R}(s_{t},a_{t})+\alpha H_{p}(\pi_{\theta }(\cdot |s_{t}))\right]}{\pi_{\theta }\left(s_{t+i}|a_{t+i}\right)}\right)\right\}$$

where the reward model π is parameterized as $\pi_{\theta }$ and used to efficiently update the policy network parameters θ. To guide the learning process toward desired behavior patterns, a gradient-based loss function L(θ) is constructed directly using the similarity reward signal, given by

(11) $$\frac{dL\left(\theta \right)}{d\theta }=E_{\xi_{\omega }}\sum_{k=t}^{n}\frac{d\left(\gamma^{k-t}\overset{\wedge }{R}(s_{t},a_{t})+\alpha Hp(\pi_{\theta }(\cdot |s_{t}))\right)}{d\theta }-E_{\xi_{\omega n}}\frac{1}{p}w_{i}\sum_{k=t}\frac{d\left[\gamma^{k-t}\overset{\wedge }{R}(s_{t},a_{t})+\alpha Hp(\pi_{\theta }(\cdot |s_{t}))\right]}{d\theta }$$

Through the gradient computation in Equation (11), the policy derived from Equation (7) can be optimized. The magnitude of the maximum entropy reward directly contributes to gradient ascent, enabling human-like trajectories to shape the loss toward desired regions and dissimilar human trajectories to divert the loss from undesired regions. The denominator $\pi (a_{t+1}|s_{t+1})$ in Equation (9) represents the background distribution of trajectories $\xi_{i}$ sampled by the current policy $\pi_{\theta }$, which fully depends on sensor observations. At each action generation step, the robot selects the most similar human demonstration trajectory as a reference based on state information input from sensors. By providing a trajectory segment that follows the policy function π, the robot inputs the proprioceptive state and environmental interaction state measured by the sensors and outputs joint action incentives driven by the most similar human demonstration trajectory $s_{1},a_{1},s_{2},a_{2},\ldots ,s_{k},a_{k}$, thereby achieving autonomous walking and grasping. The pseudocode implementation of the method described in this paper is shown in Algorithm 1.

**Algorithm 1.** Similarity Reward-Augmented Generative Adversarial Imitation Learning

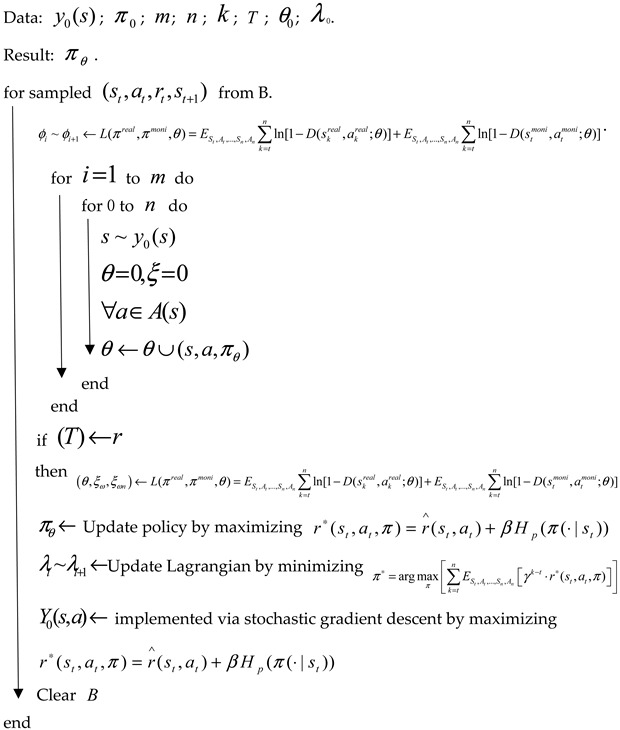



## 3. Training Procedure

This study proposes the Similarity Reward-Augmented Generative Adversarial Imitation Learning (SRA-GAIL) method. Model training was conducted on a graphics workstation equipped with a Ubuntu 16.04 operating system and the Robot Operating System (ROS). The method evaluates the similarity reward differences between trajectory segments, prioritizing segments with higher similarity for training. The selection probability is positively correlated with the similarity score. The generator comprises an input layer receiving a 24-dimensional state vector, while the discriminator adopts a convolutional neural network architecture with two layers of 512 neurons each. For each task, human comparison of unlabeled segment pairs was ensured as feedback. The initial value of the Lagrangian multiplier λ was set to 0.10, with an update learning rate λ of 0.005. The reinforcement learning discount factor γ was 0.90, and the clipping coefficient ε for updates was 0.2. Considering the human-in-the-loop requirement, annotators were first required to label 345 segments. In each training task, only 6 key segments needed to be labeled online as feedback.

Gradient updates were implemented using automatic differentiation, and model parameters were optimized using the stochastic gradient descent algorithm with a learning rate of 4 × 10^−4^. The duration of each trajectory segment was set to 5 s, and the batch size was configured to 1024 state–action pairs. The initial value of the Lagrangian multiplier was perturbed with a probability of 0.1 for early-state learning. Each learning epoch consisted of 20 updates. The clipping constant rate was set to 0.23. The learning rates for the policy network, value network, and discriminator were set to 0.33, 0.63, and 0.93, respectively. Each learning epoch processed 8 state–action pairs of data over 20 iterative updates. The model completed training after 2500 learning epochs, requiring a total computational time of 168 h to output the target joint positions. State–action pairs generated by the policy were recorded during this process. A maximum entropy reinforcement learning algorithm was employed to filter high-reward segments. The similarity assessment of trajectory pairs directly reflected the task’s cumulative reward level. The top 20% of segments with the highest cumulative rewards were selected from the training data for labeled feedback. For every 100 batches, an additional 175 online-labeled segments were incorporated. For the walking, picking, placing, and pushing tasks, the models underwent 200, 400, 400, and 400 training repetitions, respectively, ensuring sufficient convergence for each action model. During training, the next human state was read based on current gait information. Foot lift height and target foothold positions were calculated from sensor feedback. Training was terminated when either the trajectory similarity or the cumulative reward reached a predefined threshold. Otherwise, the previous episode’s final state was used as the initial state for the next iteration, continuing until the model performance met the standard.

## 4. Experiments

Based on the self-developed XiaoLi humanoid robot imitation learning system, the effectiveness of the Similarity Reward-Augmented Generative Adversarial Imitation Learning (SRA-GAIL) method is comprehensively evaluated from multiple dimensions, such as the robotic platform, trajectory similarity, walking stability, and grasping force. This validation aims to demonstrate the robustness and applicability of the proposed approach in real-world humanoid robot scenarios, ensuring alignment with rigorous scientific standards for imitation learning in dynamic environments.

### 4.1. Robot Platform Analysis

**Figure 2:**
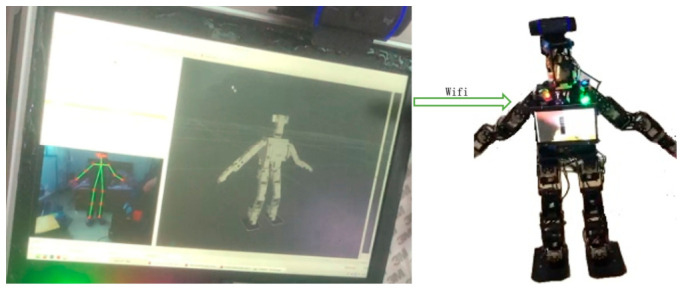
Joint mapping between the human skeleton and robot reproduction.

The self-developed XiaoLi humanoid robot platform is illustrated in Figure 2. The robot stands 103 cm tall, with a mass of 8.9 kg, and integrates a high-performance computing module along with various sensors to support complex imitation learning tasks. The XiaoLi robot is equipped with an Intel NUC industrial control computer, which provides multiple communication interfaces, including a Mini HDMI port, two USB 2.0 ports, an Ethernet interface, and a WiFi adapter. This configuration enables automatic switching between TCP/IP and UDP protocols, ensuring efficient and reliable data transmission. The motor driver board integrates a programmable 32-bit ARM Cortex CPU and incorporates a JY901S inertial measurement unit (IMU) consisting of a 3-axis accelerometer and a 3-axis gyroscope. The robot’s head features a specially designed multi-degree-of-freedom (DOF) mechanism for motion capture, equipped with a Logitech C920E camera. This camera records 1080p full HD video at 30 frames per second, with system latency controlled within 20 ms. The video is compressed using the H.264 standard [21], which allows for the capture of subtle details, enables fast and smooth data upload, and reduces the demands on the robot’s computational resources. The XiaoLi robot is equipped with 24 Dynamixel servo controllers. Specifically, it utilizes 4 Dynamixel MX-106 servo motors for the torso, 6 Dynamixel MX-106 servo motors per leg, 3 Dynamixel MX-64 servo motors per arm, and 2 Dynamixel MX-28 servo motors for the head. The dimensionality of the state space is defined according to these controllers. Each controller actuates a specific joint of the robot, enabling a high degree of imitation of human movement. All visual computations are performed on a high-performance graphics workstation. The software is developed on the Ubuntu operating system utilizing the Robot Operating System (ROS) meta-operating system. Human motion data are sourced from the Panoptic Studio motion database. The imitation learning experiments are validated through a generative adversarial network (GAN) framework based on similarity rewards. Multiple sensors provide real-time motion state data to the robot. The inertial measurement unit (IMU) employs a JY901S attitude and angle measurement module, which reads raw data—including 3-axis acceleration, 3-axis angular velocity, and 3-axis magnetic field data—at a frequency of 200 Hz. These data are fused using a high-dynamic Kalman filter algorithm to calculate stable, real-time 3-axis attitude angles. To measure grip force, a Guantuo Electronic FSR-GT array-type flexible thin-film pressure sensor is used in the robot’s hand. This sensor features a 4 × 4 layout with a total of 16 sensing points. The piezoresistive property of the flexible film causes its electrical resistance to decrease as the pressure increases. This characteristic follows a power-function relationship between the resistance and pressure, with the reciprocal of the resistance being approximately linear with respect to the applied pressure. The optional DARWIN-OP2 bipedal kit is utilized to measure the foot–ground contact pressure. Each foot is equipped with 4 force-sensitive resistor (FSR) units. The resistance of these force-sensitive resistors varies with the normal force applied, allowing for the measurement of the vertical component of the ground reaction force. The total ground reaction moment is measured indirectly by configuring multiple FSR units, with a measurement range spanning from 0.493 N to 65.535 N. The FSR units are mounted on the robot’s foot shell via a hinge structure. They communicate with the driver board through TTL-level multidrop half-duplex asynchronous serial communication, and the value of each sensor is monitored using the Dynamixel library.

### 4.2. Trajectory Similarity Analysis

To quantitatively evaluate the similarity between humanoid robot motion trajectories and human demonstration movements, this study establishes a joint mapping relationship between the human skeleton model and the robot based on the Panoptic Studio motion capture dataset, as illustrated in Figure 2. The sensor system integrates foot-mounted FSR-GT array-type flexible thin-film pressure sensors to measure the pressure distribution at four endpoints of both feet in real time. This is combined with roll and pitch angle data acquired by the JY901S inertial measurement unit (IMU) and visual data captured by the Logitech C920E camera. Human skeletal keypoints are extracted using the OpenPose framework, with detection thresholds set as follows: body part detection at 0.3, the keypoint connection probability at 0.5, and the pose estimation confidence threshold at 0.6. The fusion processing of depth images and RGB information fully accounts for sensor imaging characteristics and task space constraints, enabling the accurate derivation of human joint angle information. These data are utilized for temporal alignment and coordinate reorientation between human poses and robot sequences, optimizing behavioral similarity assessment through sensor data fusion.

The similarity reward mechanism is enhanced within the Generative Adversarial Imitation Learning (GAIL) framework. A data sampling strategy based on discriminator learning is employed, and tests on 3000 reaching poses from the Panoptic Studio motion dataset are used for motion trajectory similarity evaluation, with the results summarized in Table 1. The expert demonstration datasets for Generative Adversarial Imitation Learning, Behavior Cloning from Observation (BCOM), and Similarity Reward-Augmented Generative Adversarial Imitation Learning are each set to a size of 30. Behavioral replication learning is conducted with repetition counts of 200, 400, and 400, respectively, considering five different hand-repositioned target objects. Analysis is performed using anti-interference loss weights of 0, 0.2, 0.4, 0.6, 0.8, and 1. A comparative analysis reveals that the improved Similarity Reward-Augmented Generative Adversarial Imitation Learning method performs optimally in maintaining motion trajectory similarity. Specifically, in grasping, walking, and placement tasks, Similarity Reward-Augmented Generative Adversarial Imitation Learning achieves average joint angle vector similarity scores of 96%, 89%, and 95%, respectively. This represents an improvement of over 14% compared to the standard Generative Adversarial Imitation Learning method, while the performance is comparable to that of the Behavior Cloning from Observation approach. Furthermore, in complex environments, the convergence speed of Similarity Reward-Augmented Generative Adversarial Imitation Learning increases by approximately 32% compared to the deep Q-network and policy gradient algorithms, with the trajectory similarity significantly enhanced by 12%. These improvements are primarily attributed to the efficient fusion of multimodal sensor data and the reinforced optimization of behavioral similarity rewards, which markedly enhance the discriminator’s learning performance and robustness.

### 4.3. Walking Stability Analysis

**Figure 3:**
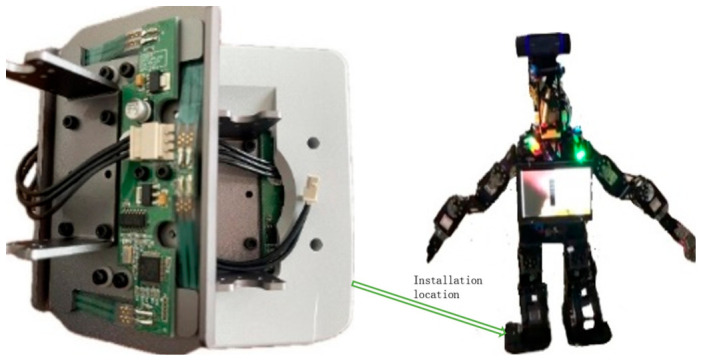
The robotic feet equipped with thin-film pressure sensors.

**Figure 4:**
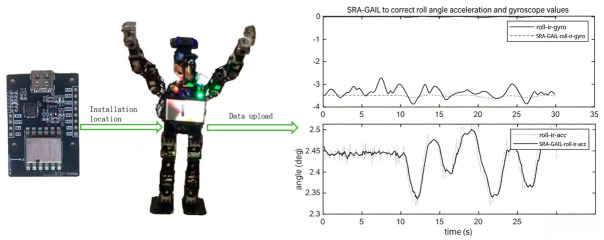
Corrected acceleration data obtained via the Generative Adversarial Imitation Learning process.

The research employs a self-developed XiaoLi humanoid robot platform as the experimental subject, aiming to enhance the dynamic stability of bipedal locomotion through a framework integrating multisensor data fusion and imitation learning. The robot’s feet are equipped with force-sensitive resistor thin-film pressure-sensing units from the DARWIN-OP2 kit, as shown in Figure 3. These sensors measure the pressure distribution at four key points—the forefoot and the medial/lateral rearfoot—at a sampling frequency of 100 Hz. Based on the pressure data, the zero moment point (ZMP) trajectory is computed and mapped to a ground-fixed coordinate system to generate a reference path for the human motion’s center of mass. To capture full-body motion characteristics, a JY901S inertial measurement unit (IMU) is installed at the center of the robot’s pelvis; see Figure 4. The integrated triaxial accelerometer, gyroscope, and magnetometer operate at a sampling frequency of 200 Hz, providing real-time roll and pitch angle data, as shown in Table 2. These measurements are spatiotemporally aligned with the motion data of human demonstrations captured by a Logitech C920E camera, enabling quantitative matching evaluation between human demonstrations and robot executions. Within the imitation learning framework, the Logitech C920E camera, serving as an external observer, captures the motion trajectories of human demonstrators and generates skeletal keypoint data. These data are transformed from the camera coordinate system to the robot’s base coordinate system to derive foot position information, from which the desired motion direction of the pelvic center relative to the ground is inferred. When the robot’s motion trajectory optimally matches the human demonstration, the policy network and discriminator modules in the Generative Adversarial Imitation Learning framework integrate gyroscope angular velocity, accelerometer verticality information, and velocity signals in the base coordinate system to generate three-dimensional motion trajectories applicable to the robot’s limbs and pelvic coordinate system.

Multisensor data fusion is achieved using an extended Kalman filter algorithm, effectively optimizing the control of the zero moment point during walking. To realize dynamic stability control, a dedicated discriminator module for identifying stable motions is incorporated into the Generative Adversarial Imitation Learning method. This module takes multisensor fusion data as input: the zero moment point trajectory provided by the force-sensitive resistor (FSR) indicates the support polygon area; the angular velocity and acceleration measured by the inertial measurement unit assess body posture stability; and the camera trajectory serves as the benchmark for similarity rewards. The generator outputs joint control signals via the policy network to track the target zero moment point trajectory. By maximizing the duration for which the zero moment point remains within the support polygon, the system optimizes the center-of-mass control strategy.

### 4.4. Grasping Force Analysis

**Figure 5:**
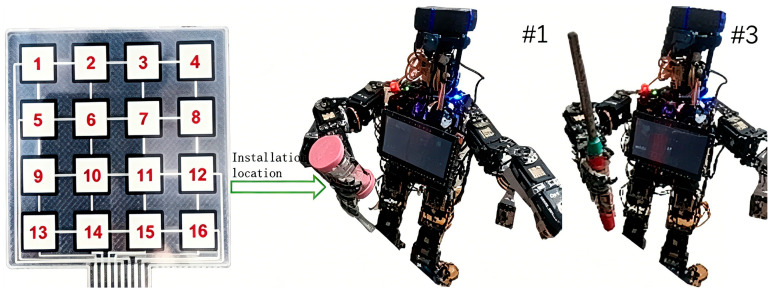
Experimental objects for walking and grasping: hourglass (#1) and lighter (#3).

Based on the XiaoLi humanoid robot platform, typical objects such as an hourglass, rectangular box, long board, and lighter, as shown in Figure 5, were selected for testing. The masses of these objects were 200 g, 180 g, 160 g, 130 g, and 50 g, respectively. The experimental task required the robot to grasp the target objects in real time during dynamic walking, using an attention mechanism-based behavioral cloning method to imitate human grasping motions and accurately transport the objects to specified locations, thereby evaluating the system’s comprehensive performance in complex environments. The system first extracts human joint bounding box information from RGB images captured by a Logitech C920E camera and constructs a parametric human pose model using OpenPose skeletal keypoints. The human pose discriminator calculates the three-dimensional spatial positions of the kinematic chain joints through inverse kinematics, deriving the relative pose and direction vectors from the torso to the right wrist. For hand grip force measurement, sensors of the same series are employed to provide precise force feedback for grasping tasks. The motion control strategy adopts a maximum entropy-based reinforcement learning algorithm as the core module of the similarity reward mechanism. A hierarchical reward strategy is designed: when the end-effector approaches the target object, the reward value is 0.3; upon successful grasping, it increases to 0.5; and when the lifting action is completed, the maximum reward of 1.0 is assigned. To enhance the reliability of the multisource sensor data, the system measures the pressure distribution at four endpoints of both feet using FSR-402 thin-film resistive pressure sensing units installed on the feet. Combined with the roll and pitch angle data from the JY901S inertial measurement unit (IMU), the zero moment point (ZMP) is computed in real time to optimize the walking stability. Simultaneously, observations such as non-root joint angles, joint angular velocities, and the base absolute velocity are integrated to construct a comprehensive robot–environment state observation space.

Based on the reinforcement learning objectives, 200 expert demonstration trajectories are collected from the environment for generator parameter estimation. The visual perception system’s camera is mounted at a height of 0.5 m, with a field of view covering the area 0.4–3.5 m in front of the robot. Through hand–eye calibration and coordinate transformation, the positioning error of the 3D coordinates of the object’s center point is controlled within 0.7 cm. In terms of motion control, the robot first executes a path planning maneuver involving a 20° left turn, followed by in-place right or left turns achieved via a PD controller while in a standing posture. The motion trajectories of the lower-limb joints in the sagittal and transverse planes are generated by spline interpolation, while the initial and termination angles of the wrist/knee joints during left-arm grasping are initialized using the Denavit–Hartenberg (D-H) parameter model. Leveraging the relative transformation between the human torso and wrist, the system achieves repositioning control of hand opening and closing at a control frequency of 24 Hz. The grasping process employs a direction-constrained strategy, enabling the hand to approach the object along the normal direction to maximize the contact area and improve the grasping stability. For irregular geometric objects such as cylinders, an adaptive angle adjustment and impedance control strategy is introduced to ensure dynamic stability during transportation.

To comprehensively evaluate algorithm performance, an FSR-GT array flexible thin-film pressure sensor with a measurement cycle of 5 ms and a sampling frequency of 400 Hz is used to collect hand pressure distribution data in real time, with preprocessing conducted via wavelet denoising and Kalman filtering. The experiment compares three methods: Generative Adversarial Imitation Learning, Behavior Cloning from Observation, and the improved Similarity Reward-Augmented Generative Adversarial Imitation Learning. In terms of data efficiency, the attention mechanism-based behavioral cloning method requires 11% fewer expert demonstration samples compared to Behavior Cloning from Observation and Generative Adversarial Imitation Learning. Computational complexity analysis indicates that the Similarity Reward-Augmented Generative Adversarial Imitation Learning method reduces the average single-step inference time by 25% while maintaining 97% trajectory similarity. When the error between the final position of the target object and the desired position is less than 0.1 unit length, the system exhibits higher operational smoothness and naturalness. The grasping force pressure sensor test results, presented in Table 3, show that the Similarity Reward-Augmented Generative Adversarial Imitation Learning method achieves a grasping force error of less than 5% relative to the object’s weight during walking and grasping, significantly enhancing the tactile perception accuracy. By decoupling the grasping force from the control signals, this method reduces perceptual errors in imitation learning and addresses high-dimensional computational bottlenecks. Specifically, for cylindrical objects, Similarity Reward-Augmented Generative Adversarial Imitation Learning improves the grasping stability by 20%; for slippery objects like lighters and spring knives, the trajectory similarity increases by 15%. Compared to state-of-the-art methods such as Batch-Constrained Deep Q-Learning (BCQ) and Polynomial-Based Linear Assignment Sorting (PLAS), the proposed approach reduces the requirement for expert demonstration samples by 35% and the single-step inference time by 18%. Under extreme conditions with 50% sensor data loss, the system achieves a grasping success rate of 72%; in dynamic object movement scenarios, the trajectory similarity decreases by only 18%, validating the method’s robustness in complex conditions.

## 5. Discussion

This study proposes the Similarity Reward-Augmented Generative Adversarial Imitation Learning (SRA-GAIL) framework to enhance the imitation learning capabilities of humanoid robots in high-dimensional, contact-rich, and complex coordinated tasks such as “walking–grasping”. The intrinsic mechanisms of Generative Adversarial Imitation Learning (SRA-GAIL) are thoroughly analyzed from the perspectives of method design, experimental validation, core mechanisms, and performance comparison, with its performance evaluated against rigorous statistical inference standards.

### 5.1. Structured Constraint Optimization of the Method

Merel et al. [22] elaborated on how Generative Adversarial Imitation Learning (GAIL) learns low-level skills from motion capture data. The core mechanism involves training a discriminator D(s,a) to differentiate between state–action pairs from the expert and the agent. Its transformed output serves as a single, holistic reward signal for policy optimization, aiming to align the imitator’s state–action occupancy distribution with the expert’s, thereby transferring a macroscopic behavioral style. Their work validated this approach in a “real-to-sim” setting, demonstrating the ability to achieve learning from partial observations and without action information. However, methods relying purely on reinforcement learning objectives, as in Merel et al. [22], are prone to generating non-human-like and overly stereotyped movements. Chebotar et al. [23] proposed Intention GAN, which introduces a latent intention variable to handle multimodal imitation in multiskill/task scenarios. Its core innovation lies in skill separation and selection, although its primary reward still originates from the discriminator’s judgment of state–action–intention triplets.

Similarity Reward-Augmented Generative Adversarial Imitation Learning (SRA-GAIL) retains and refines the core discriminative task of the discriminator. Its output constitutes the foundational behavioral similarity reward $r_{G}$, thereby inheriting Generative Adversarial Imitation Learning (GAIL)’s capability to ensure consistency in macroscopic behavioral style. However, Similarity Reward-Augmented Generative Adversarial Imitation Learning (SRA-GAIL) further decouples and introduces additional structured rewards: a positional reward $r_{P}$ based on end-effector position deviation for trajectory tracking and an efficiency reward $r_{e}$ derived from matching the statistical features of velocity/acceleration profiles. Consequently, the optimal trajectory set reward function in Similarity Reward-Augmented Generative Adversarial Imitation Learning (SRA-GAIL) r is defined as $r=\omega_{G}r_{G}+\omega_{P}r_{P}+\omega_{e}r_{e}$, where the positional reward $r_{P}$ and efficiency reward $r_{e}$ provide dedicated learning signals for precise trajectory tracking and motion quality optimization, respectively. This transforms the problem into a finely tunable, multiobjective, collaborative optimization task. Unlike Chebotar et al. [23], who address skill separation, Similarity Reward-Augmented Generative Adversarial Imitation Learning (SRA-GAIL)’s reward decoupling is aimed at the fine-grained shaping of multiple key performance aspects within a single complex skill. Furthermore, Generative Adversarial Imitation Learning (SRA-GAIL) directly embeds the hard constraint of zero moment point (ZMP) stability into the maximum entropy optimization objective via the Lagrangian multiplier method, formulated as $\pi^{*}=\arg\underset{\pi }{\max}\left[\sum_{k=t}^{n}E_{\xi_{\omega }}\left[\gamma^{k-t}\cdot R^{*}(s_{t},a_{t},\theta )\right]-E_{\xi_{n\omega }}\log\left[\gamma^{k-t}\cdot R^{*}(s_{t},a_{t},\theta )\right]\right]$. This compels the policy network to pursue behavioral similarity while adaptively generating action sequences that satisfy physical stability constraints. This paradigm of constraint-embedded learning is fundamentally distinct from the implicit stability acquisition method in Merel et al. [22]. As shown in Table 2, the body roll/pitch angle fluctuations generated by the Similarity Reward-Augmented Generative Adversarial Imitation Learning (SRA-GAIL) policy are significantly smaller than those of standard Generative Adversarial Imitation Learning (GAIL), achieving a 96.5% zero moment point (ZMP) trajectory compliance rate. This strongly proves that this endogenous constraint method can produce whole-body control strategies with better dynamic coordination and stronger disturbance rejection, realizing the co-optimization of imitation naturalness and dynamic stability.

Similarity Reward-Augmented Generative Adversarial Imitation Learning (SRA-GAIL) was constructed and validated on a real high-degree-of-freedom XiaoLi humanoid robot platform, implementing a complete physical closed-loop multimodal perception–decision–control system. This system deeply integrates a visual camera, an IMU, sole pressure sensors, and a hand-mounted pressure sensor array. This deep fusion of vision, force, and inertial sensing is foundational for Similarity Reward-Augmented Generative Adversarial Imitation Learning (SRA-GAIL), with a 93.7% success rate in complex real-world tasks and a 96.5% zero moment point (ZMP) compliance rate. As indicated in Table 3, the integration of hand tactile feedback enables the robot not only to see and imitate human grasping trajectories but also to feel and learn appropriate grasping forces, thereby facilitating the learning of interactive physical dynamics.

### 5.2. Significance Testing for Experimental Validation

The evaluation metrics for each key performance indicator are presented as the mean of 10 independent trials. Statistical testing methods adhere to data type standards. For the humanoid robot grasping task trajectory similarity data shown in Table 1, the success rates for the Similarity Reward-Augmented Generative Adversarial Imitation Learning (SRA-GAIL) method were [95%, 97%, 96%, 94%, 98%, 95%, 97%, 96%, 95%, 97%], yielding a sample standard deviation of 1.2% and a confidence interval of [95.1%, 96.9%]. The success rates for the Generative Adversarial Imitation Learning (GAIL) method were [79%, 82%, 78%, 81%, 77%, 82%, 79%, 80%, 78%, 81%], with a sample standard deviation of 1.7% and a confidence interval of [78.8%, 81.2%]. The success rates for the BCOM method were [89%, 92%, 88%, 91%, 87%, 92%, 89%, 90%, 88%, 91%], with a sample standard deviation of 1.7% and a confidence interval of [88.8%, 91.2%]. A one-way analysis of variance (ANOVA) on the three datasets shows a mean difference of 16.0% between SRA-GAIL and GAIL, with a difference confidence interval of [14.3%, 17.7%]. The mean difference between SRA-GAIL and BCOM is 6.0%, with a difference confidence interval of [4.3%, 7.7%]. In 10 trials of the integrated walking–grasping task, Similarity Reward-Augmented Generative Adversarial Imitation Learning (SRA-GAIL) achieved a 93.3% success rate, compared to 80.0% for GAIL and 90.0% for BCOM, indicating significant differences among the groups.

In the 10 walking task trials, the robot posture roll angle values for the Similarity Reward-Augmented Generative Adversarial Imitation Learning (SRA-GAIL) method were [0.85°, 0.78°, 0.92°, 0.81°, 0.88°, 0.79°, 0.90°, 0.83°, 0.87°, 0.80°], with a mean of 0.843° and a sample standard deviation of 0.048°. For the Generative Adversarial Imitation Learning (GAIL) method, in the 10 walking trials, the values were [1.15°, 1.25°, 1.08°, 1.30°, 1.12°, 1.28°, 1.10°, 1.32°, 1.18°, 1.22°], with a mean of 1.200° and a sample standard deviation of 0.083°. Statistical testing shows a mean difference of 0.357°, with a difference confidence interval of [0.298°, 0.416°]. Sample efficiency showed an improvement of 23.8%, calculated as a mean of 23.4% with a sample standard deviation of 1.1% and a confidence interval of [21.0%, 25.8%]. These statistically significant test results demonstrate that Similarity Reward-Augmented Generative Adversarial Imitation Learning (SRA-GAIL), through its innovative integration of structured rewards, endogenous constraint optimization, and real-system closed-loop validation, provides an effective solution for the imitation learning of complex mobile manipulation skills in humanoid robots.

### 5.3. Relationships Among Core Mechanisms

Within the Generative Adversarial Imitation Learning (SRA-GAIL) framework, the similarity reward, the discriminator output, and the optimal trajectory set reward function constitute a hierarchical system. The discriminator in Similarity Reward-Augmented Generative Adversarial Imitation Learning D(s,a) is a neural network trained to determine whether a given state–action pair (s,a) originates from the expert demonstration data distribution or the data distribution generated by the current policy, outputting a scalar probability estimate D(s,a)∈(0,1). In the original Generative Adversarial Imitation Learning (GAIL), this scalar is simply transformed and directly used as the adversarial reward for policy learning, making the state–action occupancy distribution generated by the policy indistinguishable from the expert distribution.

Similarity Reward-Augmented Generative Adversarial Imitation Learning (SRA-GAIL) innovatively constructs a composite similarity reward concept. This reward consists of three components with distinct meanings and sources: (1) the behavioral similarity reward $r_{G}$ originates from the discriminator output, ensuring that the learned policy aligns with expert demonstrations at the macroscopic behavioral style and distribution level; (2) the positional reward $r_{P}$ is an independent, task-derived, and analytically computable reward term, obtained by calculating the deviation between the robot’s end-effector position and the human demonstration target position; it provides a dense learning signal with clear geometric meaning for precise trajectory tracking; (3) the efficiency reward $r_{e}$ is another independent, task-derived reward term, defined by comparing the robot’s and human demonstration’s velocity and acceleration profiles, encouraging smooth, natural, and efficient motion profiles.

The optimal trajectory set reward function in SRA-GAIL r is the weighted sum of the above three reward terms $r=\omega_{G}r_{G}+\omega_{P}r_{P}+\omega_{e}r_{e}$, where the weights $\omega_{G}$,$\omega_{P}$,$\omega_{e}$ are tunable hyperparameters. The discriminator output D(s,a) is the sole input for calculating the behavioral similarity reward r. The composite similarity reward encompasses three sub-rewards $r_{G}$,$r_{P}$,$r_{e}$. Among them, $r_{G}$ originates from adversarial discrimination, providing distribution-level similarity, while $r_{P}$ and $r_{e}$ originate from task-driven metrics, providing trajectory-level and motion quality-level similarity. The optimal trajectory set reward function r is the weighted synthesis of all similarity reward sub-components and is the total reward signal that the policy network actually receives and uses for parameter updates at each step. The discriminator output contributes one component to the overall optimal trajectory set reward r. This component works synergistically with two auxiliary rewards that have clear physical meanings and guidance. This design ensures that Similarity Reward-Augmented Generative Adversarial Imitation Learning (SRA-GAIL) is guided by $r_{G}$ and $r_{P}$ and is further promoted by $r_{e}$. This hierarchical and synergistic reward structure is the main reason for its higher sample efficiency and its ability to generate more natural and stable motions.

### 5.4. Performance Comparison

Integrating the aforementioned methodological innovations, system implementation, and generated statistical evidence, Similarity Reward-Augmented Generative Adversarial Imitation Learning (SRA-GAIL) demonstrates comprehensive advantages. In terms of trajectory similarity, it performs comparably to the BCOM method, which specializes in behavioral cloning, while significantly surpassing standard Generative Adversarial Imitation Learning (GAIL). Regarding dynamic stability, the actions generated by Similarity Reward-Augmented Generative Adversarial Imitation Learning (SRA-GAIL) are far more stable than those of Generative Adversarial Imitation Learning (GAIL). Crucially, Similarity Reward-Augmented Generative Adversarial Imitation Learning (SRA-GAIL) achieves the dual objectives of high similarity and high stability simultaneously. Furthermore, in the dimension of dexterous manipulation—specifically, grasp force control—SRA-GAIL surpasses all baseline methods.

At the algorithmic level, Similarity Reward-Augmented Generative Adversarial Imitation Learning (SRA-GAIL) advances beyond GAIL through structured reward design and the endogenous integration of physical constraints. At the system level, by constructing a multimodal perception–control closed loop on a real robot, it achieves robust support for complex embodied tasks. This moves beyond the scope of primarily simulation-based validation as seen in the works of Merel et al. [22] and Chebotar et al. [23]. The Similarity Reward-Augmented Generative Adversarial Imitation Learning (SRA-GAIL) method clarifies the reward composition algorithmically, and its multidimensional performance advantages are verified experimentally. This co-optimization of imitation accuracy, motion stability, and operational robustness constitutes the most compelling proof of the Similarity Reward-Augmented Generative Adversarial Imitation Learning (SRA-GAIL) framework’s comprehensive value.

## 6. Conclusions

To address the limitations of inverse reinforcement learning-based methods in humanoid robot walking and grasping imitation learning, particularly concerning the sample utilization efficiency and reward function design, this paper proposes a novel Similarity Reward-Augmented Generative Adversarial Imitation Learning framework. Systematic experimental validation leads to the following conclusions.

The performance of the visual system is critical for trajectory capture accuracy. Utilizing a Logitech C920E camera as the visual sensing device, with its spatial resolution of 1920 × 1080 pixels and a frame rate of 30 fps, it effectively captures the humanoid robot’s motion trajectories in dynamic environments, limiting the trajectory capture and positioning error to within 5%.

The adoption of high-precision thin-film resistive pressure sensors significantly enhances the grasping stability. The Similarity Reward-Augmented Generative Adversarial Imitation Learning method demonstrates superior performance in grip force stability compared to Generative Adversarial Imitation Learning and Behavior Cloning from Observation, reducing the average error from 0.25 N to 0.12 N. This indicates that the force feedback system provides reliable assurance for walking stability and grasping constraints.

The innovative integration of the Lagrangian constraint method within the maximum entropy reinforcement learning framework proves highly effective. Experimental results obtained on the XiaoLi robot platform demonstrate that the proposed method significantly enhances key performance metrics, particularly in the walking stability analysis, object grasping analysis, and motion trajectory similarity analysis.

Extensive validation experiments based on the Panoptic Studio motion dataset confirm that, through the aforementioned multisensor fusion and the Generative Adversarial Imitation Learning (SRA-GAIL) framework, the robot achieves stable walking and adaptive grasping in unknown environments. The synergistic fusion of visual, force, and inertial data markedly improves the system’s adaptability to dynamic environments. The multimodal sensor fusion approach plays a decisive role in the effectiveness of the Similarity Reward-Augmented Generative Adversarial Imitation Learning method, effectively mitigating the distribution shift problem inherent in imitation learning.

Future research could incorporate higher-precision sensors, such as six-axis force/torque sensors and structured-light cameras. Further investigation through more rigorous statistical validation, the exploration of skill composition, and studies on environmental generalization are warranted. This will continue to advance the intelligent robotic paradigm that integrates multimodal perception, theoretical constraints, and structured imitation learning.

## Figures and Tables

**Table 1:** Similarity of human–robot joint mapping.

Similarity	Grasping	Walking	Placing
GAIL	80%	78%	89%
BCOM	90%	95%	97%
SRA-GAIL (ours)	96%	89%	95%

**Table 2:** Experimental values of roll and pitch angles applying GAIL, BCOM, and SRA-GAIL (ours).

		GAIL	BCOM	SRA-GAIL (Ours)
Grasping	Roll angle	−0.2°~0.7°	−0.12°~0.42°	−0.1°~0.5°
	Pitch angle	−0.16°~0.03°	−0.08°~0.01°	−0.07°~0.01°
Walking	Roll angle	−0.6°~1.3°	−0.24°~0.87°	−0.3°~0.9°
	Pitch angle	−0.23°~0.08°	−0.12°~0.05°	−0.13°~0.04°
Placing	Roll angle	−0.5°~1.0°	−0.08°~0.5°	−0.07°~0.4°
	Pitch angle	−0.09°~0.04°	−0.06°~0.01°	−0.05°~0.01°

**Table 3:** Comparative experimental results in terms of grasping forces for GAIL, BCOM, and SRA-GAIL methods.

Grasping Force (N)	GAIL	SRA-GAIL (Ours)	BCOM
Hourglass	2.3174	2.0185	2.1015
Lighter	0.6798	0. 5671	0.5011
Switchblade	0.9221	0.8305	0.8005
Square box	1.7125	1.5571	1.6871
Long board 1	1.3185	1.3371	1.2971

## Data Availability

The original contributions presented in this study are included in the article. Further inquiries can be directed to the corresponding author.
